# The impact of fluid status and decremental PEEP strategy on cardiac function and lung and kidney damage in mild-moderate experimental acute respiratory distress syndrome

**DOI:** 10.1186/s12931-021-01811-y

**Published:** 2021-07-30

**Authors:** Nazareth N. Rocha, Cynthia S. Samary, Mariana A. Antunes, Milena V. Oliveira, Matheus R. Hemerly, Patrine S. Santos, Vera L. Capelozzi, Fernanda F. Cruz, John J. Marini, Pedro L. Silva, Paolo Pelosi, Patricia R. M. Rocco

**Affiliations:** 1 Laboratory of Pulmonary Investigation, Carlos Chagas Filho Institute of Biophysics, Federal University of Rio de Janeiro, Rio de Janeiro, Brazil; 2 Department of Physiology and Pharmacology, Biomedical Institute, Niteroi, Brazil; 3 Department of Physiotherapy, Faculty of Medicine, Federal University of Rio de Janeiro, Rio de Janeiro, Brazil; 4 Department of Pathology, School of Medicine, University of São Paulo, São Paulo, Brazil; 5 Division of Pulmonary and Critical Care Medicine, Regions Hospital, University of Minnesota, St. Paul, MN USA; 6 Department of Surgical Sciences and Integrated Diagnostics (DISC), University of Genoa, Genoa, Italy; 7 San Martino Policlinico Hospital, IRCCS for Oncology and Neurosciences, Genoa, Italy; 8 Laboratory of Pulmonary Investigation, Instituto de Biofísica Carlos Chagas Filho, Universidade Federal do Rio de Janeiro, Centro de Ciências da Saúde, Avenida Carlos Chagas Filho, s/n, Bloco G-014, Ilha do Fundão, Rio de Janeiro, RJ 21941-902 Brazil

**Keywords:** Mechanical ventilation, PEEP, Inflammation, Fluids, Kidney, Heart

## Abstract

**Background:**

We evaluated the effects of abrupt versus gradual PEEP decrease, combined with standard versus high-volume fluid administration, on cardiac function, as well as lung and kidney damage in an established model of mild-moderate acute respiratory distress syndrome (ARDS).

**Methods:**

Wistar rats received endotoxin intratracheally. After 24 h, they were treated with Ringer’s lactate at standard (10 mL/kg/h) or high (30 mL/kg/h) dose. For 30 min, all animals were mechanically ventilated with tidal volume = 6 mL/kg and PEEP = 9 cmH_2_O (to keep alveoli open), then randomized to undergo abrupt or gradual (0.2 cmH_2_O/min for 30 min) PEEP decrease from 9 to 3 cmH_2_O. Animals were then further ventilated for 10 min at PEEP = 3 cmH_2_O, euthanized, and their lungs and kidneys removed for molecular biology analysis.

**Results:**

At the end of the experiment, left and right ventricular end-diastolic areas were greater in animals treated with high compared to standard fluid administration, regardless of PEEP decrease rate. However, pulmonary arterial pressure, indicated by the pulmonary acceleration time (PAT)/pulmonary ejection time (PET) ratio, was higher in abrupt compared to gradual PEEP decrease, independent of fluid status. Animals treated with high fluids and abrupt PEEP decrease exhibited greater diffuse alveolar damage and higher expression of interleukin-6 (a pro-inflammatory marker) and vascular endothelial growth factor (a marker of endothelial cell damage) compared to the other groups. The combination of standard fluid administration and gradual PEEP decrease increased zonula occludens-1 expression, suggesting epithelial cell preservation. Expression of club cell-16 protein, an alveolar epithelial cell damage marker, was higher in abrupt compared to gradual PEEP decrease groups, regardless of fluid status. Acute kidney injury score and gene expression of kidney injury molecule-1 were higher in the high versus standard fluid administration groups, regardless of PEEP decrease rate.

**Conclusion:**

In the ARDS model used herein, decreasing PEEP abruptly increased pulmonary arterial hypertension, independent of fluid status. The combination of abrupt PEEP decrease and high fluid administration led to greater lung and kidney damage. This information adds to the growing body of evidence that supports gradual transitioning of ventilatory patterns and warrants directing additional investigative effort into vascular and deflation issues that impact lung protection.

**Supplementary Information:**

The online version contains supplementary material available at 10.1186/s12931-021-01811-y.

## Background

Lung-protective strategies aimed at preventing ventilator-induced lung injury (VILI) have focused strictly on the regulation of airspace pressures, excursions, and frequencies [1]. By comparison, events affecting the opposite (vascular) side of the gas-exchanging membrane have been relatively neglected [1]. Yet, there is ample reason to conclude that events within the gas and vascular spaces interact in VILI pathogenesis. Convincing experimental evidence indicates that microvascular pressures and flows strongly influence lung edema and VILI expression [2, 3]. Moreover, fluid balance, a presumed correlate of vessel filling, relates directly to clinical outcomes for as yet incompletely defined reasons [4]. As noted in previous work by our group [5, 6], the *rates* at which ventilatory interventions are imposed or withdrawn may also contribute to VILI risk. Gradual increases in tidal volume (V_T_) [7] PEEP [8] and recruiting pressures [9, 10] appear to be better tolerated than sudden ones, presumably by allowing a more beneficial distribution of stress and strain. On the deflation side, data gathered in recent years from small-animal models with previously healthy lungs demonstrate the adverse influence of abruptly releasing high levels of PEEP [11]. This observation has been attributed primarily to cardiovascular compromise owing to the initial “surge” of translocating fluid volumes from peripheral to central vascular compartments. In experimental acute respiratory distress syndrome (ARDS); however, alveoli have different time constants and lung tissue sensitivity to vascular volume and flows, due to atelectasis and edema. In theory, the lung may be influenced not only by deflation kinetics, but also by the pace of PEEP release.

Fluid administration, like any other treatment, might induce significant side effects in experimental ARDS. Experimental [12] and clinical [13] studies reported that reducing lung vascular hydrostatic pressures decreases lung edema in ARDS, likely due to favorable effects on Starling forces and attenuated lung inflammation [14]. Despite a considerable body of published evidence that implicates the importance of fluid volume status and *ventilatory* transitions in VILI and organ dysfunction, to our knowledge, the relative effects of abrupt as opposed to gradual release of PEEP on the lung, heart, and other vital organs have not been investigated. Thus, the aim of the present study was to evaluate the effects of abrupt versus gradual PEEP release—combined with standard or high fluid volumes—on the cardiac function, as well as lung and kidney damage in an established animal model of ARDS.

## Material and methods

### Study approval

This study was approved by the Animal Care and Use Committee of the Health Sciences Center, Federal University of Rio de Janeiro, Rio de Janeiro, Brazil, with opinion number 122/18. All animals received humane care in compliance with the “Principles of Laboratory Animal Care” formulated by the National Society for Medical Research and the U.S. National Academy of Sciences *Guide for the Care and Use of Laboratory Animals*. Animals were housed at a controlled temperature (23 °C) and controlled light–dark cycle (12–12 h), with free access to water and food.

### Animal preparation and experimental protocol

**Fig. 1:**
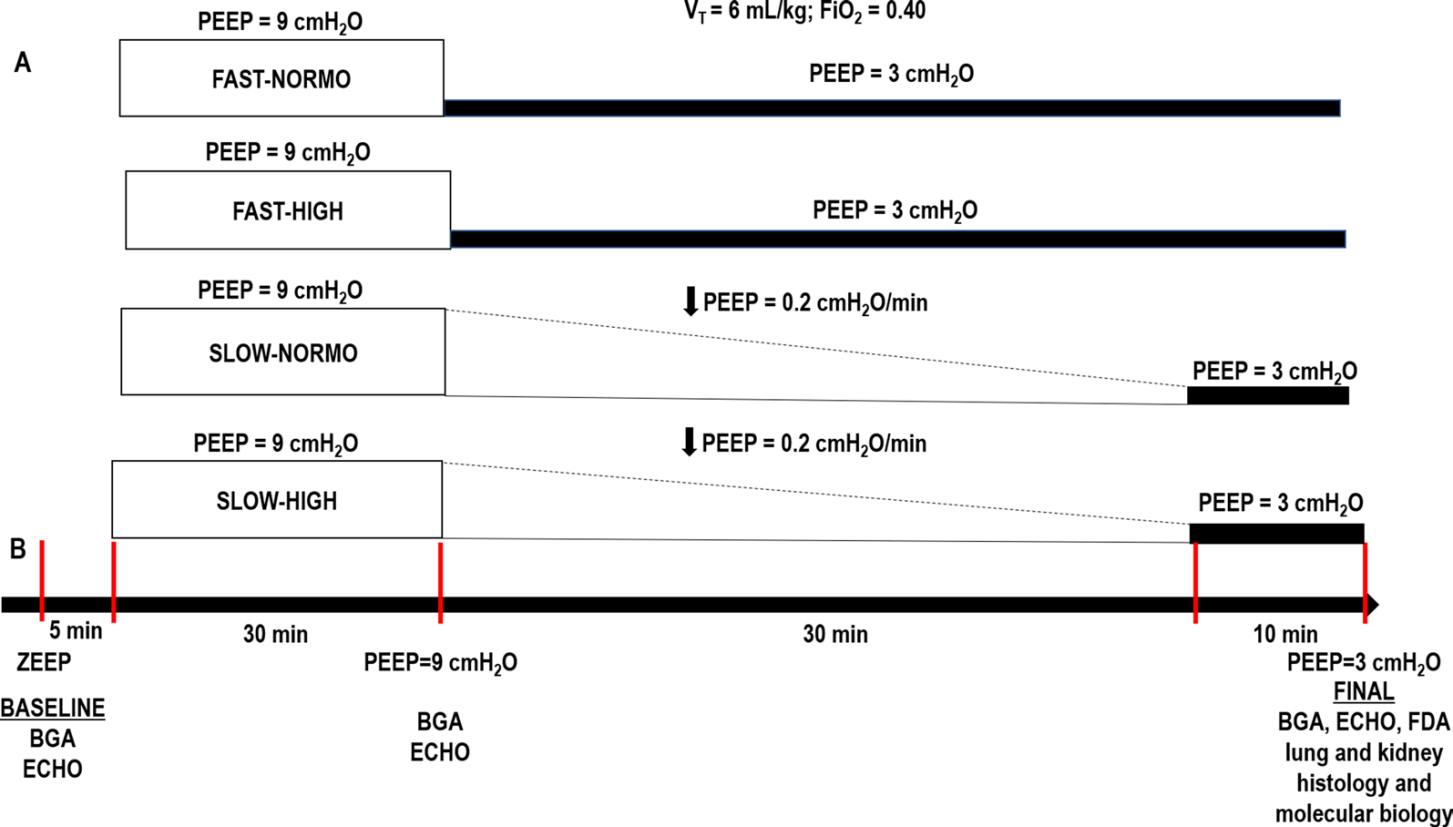
Protocol (**A**) and timeline representation of the experimental protocol (**B**). Arterial blood gases and echocardiography were evaluated at BASELINE and FINAL. *BGA* blood gas analysis; *ECHO* echocardiography; *FDA* functional data acquisition; *FiO*_*2*_ fraction of inspired oxygen; *ZEEP* zero end-expiratory pressure; *PEEP* positive end-expiratory pressure; *V*_*T*_ tidal volume. Twenty-four hours after intratracheal administration of *Escherichia coli* lipopolysaccharide, rats received standard (10 mL/kg/h, NORMO) or high (30 mL/kg/h, HIGH) volume of Ringer’s lactate. For 30 min, all rats, regardless of fluid status, were mechanically ventilated with V_T_ = 6 mL/kg and PEEP = 9 cmH_2_O, and then randomized to the following groups: abrupt PEEP decrease from 9 to 3 cmH_2_O (FAST) or gradual PEEP decrease (0.2 cmH_2_O/min) from 9 to 3 cmH_2_O (SLOW) for 30 min

Thirty-five male Wistar rats (age 8–10 weeks, body weight 291 ± 75 g) were used. Rats were anesthetized by inhalation of 1.0% sevoflurane (Sevorane®; Cristália, Itapira, SP, Brazil) and received *Escherichia coli* lipopolysaccharide (LPS: 9.6 × 10^6^ EU/mL in 200 μL of saline solution) intratracheally (i.t.) to induce experimental acute respiratory distress syndrome [7] Twenty-four hours after ARDS induction, animals were premedicated intraperitoneally (i.p.) with 10 mg/kg diazepam (Compaz®, Cristália, Itapira, SP, Brazil), followed by 100 mg/kg ketamine (Ketamin-S®, Cristália, Itapira, SP, Brazil) and 2 mg/kg midazolam (Dormicum®, União Química, São Paulo, SP, Brazil). After local anesthesia with 2% lidocaine (0.4 mL), a midline neck incision and tracheostomy were performed. An intravenous (i.v.) catheter (Jelco 24G, Becton, Dickinson and Company, New Jersey, NJ, USA) was inserted into the tail vein, and anesthesia induced and maintained with midazolam (2 mg/kg/h) and ketamine (50 mg/kg/h). A second catheter (18G, Arrow International, USA) was then placed in the right internal carotid artery for blood sampling and gas analysis (Radiometer ABL80 FLEX, Copenhagen NV, Denmark), as well as monitoring of mean arterial pressure (MAP) (Networked Multiparameter Veterinary Monitor LifeWindow 6000V; Digicare Animal Health, Boynton Beach, FL, USA). Heart rate (HR), MAP, and rectal temperature were continuously monitored (Networked Multiparameter Veterinary Monitor LifeWindow 6000V, Digicare Animal Health, Florida, USA). Body temperature was maintained at 37.5 ± 1 °C using a heating bed. Animals in dorsal recumbency were paralyzed with pancuronium bromide (2 mg/kg, i.v.) and their lungs mechanically ventilated (V500; Dräger Medical, Lübeck, Germany) in volume-controlled mode (VCV) with constant inspiratory airflow, V_T_ = 6 mL/kg, respiratory rate (RR) to maintain V′_E_ = 160 mL/min, zero end-expiratory pressure (ZEEP), FiO_2_ = 1.0, and an inspiratory-expiratory ratio of 1:2 (BASELINE). Arterial blood gases and echocardiography were evaluated. PEEP was then progressively (over 5 min) increased to 9 cmH_2_O, while, at the same time, animals were randomized to receive a standard (10 mL/kg, NORMO) or high (30 mL/kg/h, HIGH) volume of Ringer’s lactate (B. Braun, Crissier, Switzerland) via continuous intravenous infusion until the end of the study. For 30 min, all animals were mechanically ventilated with tidal volume = 6 mL/kg and PEEP = 9 cmH_2_O (to keep alveoli open), then randomized to undergo abrupt or gradual (0.2 cmH_2_O/min for 30 min) PEEP decrease, from 9 to 3 cmH_2_O (Fig. 1). After this period, animals were further ventilated for 10 min at PEEP = 3 cmH_2_O. The PEEP levels of 3 and 9 cmH_2_O are often used in rats. Theoretical analyses have shown that PEEP levels in rats could be equivalent to double those in humans, according to the estimated transpulmonary pressure [13]. Therefore, the range of PEEP levels used in the current study was selected to resemble those used in mechanically ventilated critical-care patients (6–18  cmH_2_O). At the end of the experiment, arterial blood gases, echocardiography, and respiratory system mechanics were assessed (FINAL), and heparin (1000 IU) was injected into the tail vein. All animals were killed by overdose of sodium thiopental (60 mg/kg i.v.) and the trachea was then clamped at PEEP = 3 cmH_2_O. Lungs and kidney were then extracted for histology and molecular biology analysis. Seven animals received LPS intratracheally but were not mechanically ventilated [non-ventilated (NV) animals]; after 24 h, they were euthanized and had their lungs and one kidney removed for molecular biology analysis.

### Data acquisition and processing

Airflow and airway pressure were continuously recorded throughout the experiments [7, 15, 16]. V_T_, RR, and V′_E_ were calculated. Respiratory system mechanics were assessed by occluding the airways at end-inspiration for 5 s until a respiratory system plateau pressure (Pplat,_RS_) was reached. Respiratory system driving pressure (∆P,_RS_) was calculated as the difference between Pplat,_RS_ (post end-inspiratory pause) and PEEP. All signals were amplified in a four-channel signal conditioner (SC-24, SCIREQ, Montreal, QC, Canada), and sampled at 200 Hz with a 12-bit analog-to-digital converter (National Instruments; Austin, Texas, USA). Mechanical data were computed offline by a routine written in MATLAB (Version R2007a; The Mathworks Inc., Natick, Massachusetts, USA).

### Transthoracic echocardiography

Shaved animals were placed in the dorsal recumbent position. Transthoracic echocardiography was performed by an expert (N.N.R.) blinded to group allocation, using an UGEO HM70A system (Samsung, São Paulo, Brazil) equipped with a linear phased-array probe (8–13 MHz). Images were obtained from the subcostal and parasternal views. Transthoracic echocardiography was performed [17] and the following parameters analyzed: right ventricular (RV) area, left ventricular (LV) area, and right ventricular cardiac output (RVCO). Pulsed-wave Doppler was used to measure the ratio of pulmonary acceleration time (PAT, time from the onset of pulmonary flow to peak velocity by pulsed-wave Doppler recording) to pulmonary ejection time (PET, time interval between the onset and end of the systolic flow velocity), which is an indirect index of pulmonary arterial hypertension [18]. All parameters followed American Society of Echocardiography and European Association of Cardiovascular Imaging recommendations [17].

### Histology

#### Diffuse alveolar damage

The lungs and heart were removed en bloc*.* The left lung was frozen in liquid nitrogen and immersed in formaldehyde solution (4%), embedded in paraffin, cut longitudinally in the central zone by means of a microtome into three slices (each 4 μm thick), and stained with hematoxylin–eosin for histological analysis [7, 15]. Photomicrographs at magnifications of × 100, × 200, and × 400 were obtained from eight non-overlapping fields of view per section using a light microscope (Olympus BX51, Olympus Latin America Inc., Brazil). Diffuse alveolar damage (DAD) was quantified using a weighted scoring system by two investigators (M.V. and V.L.C.) blinded to group assignment, working independently, as described elsewhere [19]. Briefly, scores of 0–4 were used to represent interstitial edema, overdistension, alveolar collapse, septal inflammation, and alveolar hemorrhage, with 0 standing for no effect and 4 for maximum severity. Additionally, the extent of each scored characteristic per field of view was determined on a scale of 0–4, with 0 standing for no visible evidence and 4 for complete involvement. Scores were calculated as the product of severity and extent of each feature, on a range of 0–16. The cumulative DAD score was calculated as the sum of each score characteristic and ranged from 0 to 80, as described elsewhere [19].

#### Acute kidney injury score

Kidney slices were stained with hematoxylin–eosin and periodic acid–Schiff and observed under light microscopy for qualitative and quantitative analysis. Semiquantitative data were obtained from high-resolution photomicrographs. Fifteen non-overlapping images of tubular tissue (cortex and outer medulla) were randomly obtained with a × 40 objective lens from each kidney section (n = 8/group) stained with H&E and PAS (tubular profiles). Histological findings were graded from 0 to 4 (0, no change; 1, changes affecting 25% of the field of view; 2, changes affecting 25–50%; 3, changes affecting 51–75%, and 4, changes affecting > 75% of the field), according to the area affected by the features of interest (edema, tubular cell vacuolization, deranged brush border in proximal tubular epithelia, tubular cell death/desquamation, and inflammation). The final kidney injury score in each rat was expressed as the sum of all values of all features obtained and ranged from 0 to 20 [20].

#### Transmission electron microscopy

Three slices (2 × 2 × 2 mm) were cut from three different segments of the left lung and fixed in 2.5% glutaraldehyde for electron microscopy. On each lung electron microscopy image (20 fields per animal), degree of interstitial edema, damage to basement membrane, extracellular matrix damage, type II epithelial cell damage, and endothelial cell damage were graded on a five-point, semiquantitative, severity-based scoring system as follows: 0 = normal lung parenchyma, 1–4 = changes in 1–25%, 26–50%, 51–75%, and 76–100% of examined tissue, respectively [21]. All histological analyses were performed in a blinded manner.

#### Biological markers

Quantitative real-time reverse transcription polymerase chain reaction (RT-PCR) was used. In lung tissue, gene expression of biomarkers associated with inflammation (interleukin [IL]-6), tight junction (zona occludens [ZO]-1), epithelial cell damage (club cell secretory protein 16, CC16), extracellular matrix damage (versican and syndecan-1), and endothelial cell damage (vascular endothelial growth factor, VEGF) were measured. In kidney tissue, gene expressions of biomarkers associated with renal injury (kidney injury molecule [KIM]-1 and neutrophil gelatinase associated lipocalin, NGAL) and inflammation (IL-6) were evaluated. The primer sequences are listed in Additional file 1: Table S1. Central slices of the right lung and kidney were cut, flash-frozen by immersion in liquid nitrogen, and stored at − 80 °C. For each sample, the expression of each gene was normalized to the acidic ribosomal phosphoprotein P0 (*36B4*) housekeeping gene [22] and expressed as fold change relative to NV group, using the 2^−∆∆Ct^ method, where ΔCt = Ct (target gene) – Ct (reference gene) [23].

### Statistical analysis

Sample size was calculated on the basis of pilot studies, which detected differences in IL-6 between abrupt and gradual PEEP decrease under high fluid administration. A sample size of 7 rats per group would provide the appropriate power (1 − β = 0.8) to identify significant differences in IL-6 expression, taking into account the effect size d = 2.0, a two-sided *t* test, and a sample size ratio of 1 (G*Power 3.1.9.2., University of Dusseldorf, Dusseldorf, Germany). Normality and equality of variance were evaluated by the Kolmogorov–Smirnov test with Lilliefors’ correction and Levene’s median test, respectively. Two-way ANOVA followed by Tukey’s test was used to compare abrupt and gradual PEEP release under standard and high fluid volume conditions. Parametric data were expressed as mean ± SD, while nonparametric data were expressed as median (interquartile range). All tests were carried out in GraphPad Prism 8.00 (GraphPad Software, La Jolla, CA, USA). Significance was established at p < 0.05.

## Results

No mortality was observed in any group during the experiments. At FINAL, the mean volumes of fluids injected were 6.9 ± 2.5 mL in the group treated with standard fluid administration (NORMO) with abrupt PEEP decrease (FAST), 16.1 ± 7.2 mL in the high fluid administration (HIGH) with abrupt PEEP decrease group, 6.0 ± 1.5 mL in the standard fluid administration with gradual PEEP decrease (SLOW) group, and 15.4 ± 1.9 mL in high fluid administration with gradual PEEP decrease group.

At BASELINE, echocardiographic parameters did not differ among groups. At FINAL, high fluid administration resulted in increased LV and RV areas, as well as RVCO, regardless of velocity of PEEP decrease (Table 1). Compared to gradual PEEP decrease, abrupt deflation reduced PAT/PET independent of fluid status, suggesting higher pulmonary arterial pressures (Additional file 2: Fig. S1). Heart rate did not differ between groups, and mean arterial pressure remained above 70 mmHg throughout the experiments.

**Table 1:** Echocardiographic parameters at ZEEP (BASELINE), after PEEP = 9 cmH_2_O, and at FINAL

	FLUIDS	PEEP	ZEEP	PEEP 9	FINAL
PAT/PET	NORMO	FAST	0.38 ± 0.06	0.36 ± 0.08	0.31 ± 0.10
	HIGH		0.38 ± 0.03	0.41 ± 0.08	0.34 ± 0.10
	NORMO	SLOW	0.40 ± 0.11	0.39 ± 0.13	0.47 ± 0.05^#^
	HIGH		0.40 ± 0.07	0.42 ± 0.08	0.48 ± 0.05*
LV area (cm^2^)	NORMO	FAST	0.22 ± 0.09	0.18 ± 0.07	0.20 ± 0.04*
	HIGH		0.25 ± 0.13	0.25 ± 0.08	0.38 ± 0.09
	NORMO	SLOW	0.19 ± 0.03	0.23 ± 0.09	0.18 ± 0.07**
	HIGH		0.23 ± 0.10	0.18 ± 0.06	0.30 ± 0.08
RV area (cm^2^)	NORMO	FAST	0.32 ± 0.05	0.32 ± 0.07	0.29 ± 0.03*
	HIGH		0.33 ± 0.08	0.36 ± 0.04	0.43 ± 0.11
	NORMO	SLOW	0.32 ± 0.08	0.31 ± 0.05	0.30 ± 0.05**
	HIGH		0.33 ± 0.07	0.28 ± 0.09	0.34 ± 0.03
RVCO (L/min)	NORMO	FAST	0.12 ± 0.04	0.14 ± 0.02	0.09 ± 0.04*
	HIGH		0.12 ± 0.03	0.15 ± 0.10	0.19 ± 0.07
	NORMO	SLOW	0.11 ± 0.03	0.14 ± 0.05	0.06 ± 0.02**
	HIGH		0.13 ± 0.06	0.14 ± 0.09	0.16 ± 0.06
HR (bpm)	NORMO	FAST	341 ± 52	352 ± 67	330 ± 85
	HIGH		371 ± 47	334 ± 64	324 ± 59
	NORMO	SLOW	361 ± 43	337 ± 23	339 ± 31
	HIGH		375 ± 48	349 ± 23	353 ± 48
MAP (mmHg)	NORMO	FAST	118 ± 26	116 ± 36	127 ± 40
	HIGH		115 ± 33	109 ± 38	117 ± 48
	NORMO	SLOW	112 ± 21	111 ± 28	105 ± 47
	HIGH		121 ± 13	107 ± 35	108 ± 33

Values are means ± SD of 7 animals/group. All data were collected before fluid administration and in ZEEP (BASELINE), after PEEP of 9 cmH_2_O and at FINAL. NORMO and HIGH: administration of Ringer lactate at 10 mL/kg/h and 30 mL/kg/h, respectively. FAST: abrupt PEEP decrease from 9 to 3 cmH_2_O. SLOW: gradual PEEP decrease (0.2 cmH_2_O/min) from 9 to 3 cmH_2_O

*PAT* pulmonary acceleration time, *PET* pulmonary ejection time, *PAT/PET ratio* indirect index of pulmonary arterial hypertension, *LV area* left ventricular end-diastolic area, *RV area* right ventricular end-diastolic area, *RVCO* right ventricular cardiac output, *HR* heart rate, *MAP* mean arterial pressure

*Significantly different from HIGH-FAST (p < 0.05); **Significantly different from HIGH-FAST (p < 0.01)

^#^Significantly different from NORMO-FAST (p < 0.0125). ^#^Significantly different from HIGH-SLOW (p < 0.05)

Arterial blood gases did not differ at BASELINE, suggesting similar degrees of lung damage. Oxygenation improved both at PEEP of 9 cmH_2_O and at FINAL, whereas PaCO_2_ was higher in FINAL compared to BASELINE. At FINAL, pHa, PaO_2_, PaCO_2_, and HCO_3_^−^ did not differ among groups (Table 2).

**Table 2:** Arterial blood gases at ZEEP (BASELINE), after PEEP = 9 cmH_2_O and at FINAL

	FLUIDS	PEEP	ZEEP	PEEP 9	FINAL
pHa	NORMO	FAST	7.24 ± 0.09	7.19 ± 0.23	7.16 ± 0.09
	HIGH		7.25 ± 0.04	7.18 ± 0.08	7.17 ± 0.07
	NORMO	SLOW	7.23 ± 0.05	7.21 ± 0.20	7.14 ± 0.08
	HIGH		7.23 ± 0.06	7.13 ± 0.10	7.15 ± 0.08
PaO_2_/FiO_2_ (mmHg)	NORMO	FAST	241 ± 114	435 ± 40*	390 ± 51*
	HIGH		232 ± 90	365 ± 55*	425 ± 35*
	NORMO	SLOW	222 ± 70	430 ± 25*	396 ± 31*
	HIGH		282 ± 89	390 ± 95*	360 ± 55*
PaCO_2_ (mmHg)	NORMO	FAST	45 ± 6	59 ± 9*	77 ± 22*
	HIGH		48 ± 5	58 ± 8*	62 ± 17
	NORMO	SLOW	50 ± 6	63 ± 8	65 ± 12
	HIGH		51 ± 5	59 ± 10	68 ± 14*
HCO_3_^−^ (mEq/L)	NORMO	FAST	20 ± 4	20 ± 5	25 ± 5
	HIGH		22 ± 6	22 ± 7	23 ± 6
	NORMO	SLOW	21 ± 6	21 ± 6	21 ± 4
	HIGH		23 ± 7	21 ± 7	24 ± 7

Values are means ± SD of 7 animals/group. NORMO and HIGH: administration of Ringer lactate at 10 mL/kg/h and 30 mL/kg/h, respectively. FAST: abrupt PEEP decrease from 9 to 3 cmH_2_O. SLOW: gradual PEEP decrease (0.2 cmH_2_O/min) from 9 to 3 cmH_2_O

*pHa* arterial pH, *PaO*_*2*_*/FiO*_*2*_ ratio of partial pressure of oxygen in arterial blood to fraction of inspired oxygen, *PaCO*_*2*_ partial pressure of carbon dioxide, *HCO*_*3*_^*−*^ bicarbonate

*Significantly different from BASELINE (p < 0.05)

At FINAL, V_T_, RR, V′_E_, and Ti/Ttot (Table 3) did not differ between groups. In animals treated with high fluid administration, Pplat,_RS_ and ∆P,_RS_ were higher in FAST compared to NORMO groups (Table 3). In the presence of standard fluid administration, no significant differences were observed in Pplat,_RS_ or ∆P,_RS_ between FAST and NORMO groups.

**Table 3:** Respiratory parameters at FINAL

	FLUIDS	PEEP	FINAL
V_T_ (mL/kg)	NORMO	FAST	6.1 ± 1.2
	HIGH		6.4 ± 1.0
	NORMO	SLOW	6.0 ± 1.0
	HIGH		6.5 ± 0.6
RR (bpm)	NORMO	FAST	113 ± 25
	HIGH		104 ± 38
	NORMO	SLOW	120 ± 29
	HIGH		100 ± 24
V′_E_ (mL/min)	NORMO	FAST	202 ± 28
	HIGH		203 ± 37
	NORMO	SLOW	206 ± 17
	HIGH		205 ± 24
Ti/Ttot (s)	NORMO	FAST	0.4 ± 0.1
	HIGH		0.4 ± 0.0
	NORMO	SLOW	0.3 ± 0.0
	HIGH		0.4 ± 0.0
Pplat,_RS_ (cmH_2_O)	NORMO	FAST	13.3 ± 4.7
	HIGH		15.1 ± 2.7
	NORMO	SLOW	10.7 ± 1.2
	HIGH		10.8 ± 1.3*
∆P,_RS_ (cmH_2_O)	NORMO	FAST	9.9 ± 2.9
	HIGH		12.7 ± 2.5
	NORMO	SLOW	8.9 ± 1.5
	HIGH		8.5 ± 1.3*

Values are means ± SD of 7 animals/group. Respiratory variables obtained at FINAL. NORMO and HIGH: administration of Ringer lactate at 10 mL/kg/h and 30 mL/kg/h, respectively. FAST: abrupt PEEP decrease from 9 to 3 cmH_2_O. SLOW: gradual PEEP decrease (0.2 cmH_2_O/min) from 9 to 3 cmH_2_O

*V*_*T*_ tidal volume, *RR* respiratory rate, *V′*_*E*_ minute ventilation, *Ti/Ttot* ratio of inspiratory time to total time, *Pplat,*_*RS*_ respiratory system plateau pressure, ∆*P,*_*RS*_ respiratory system driving pressure

*Significantly different from HIGH-FAST (p < 0.0125)

**Fig. 2:**
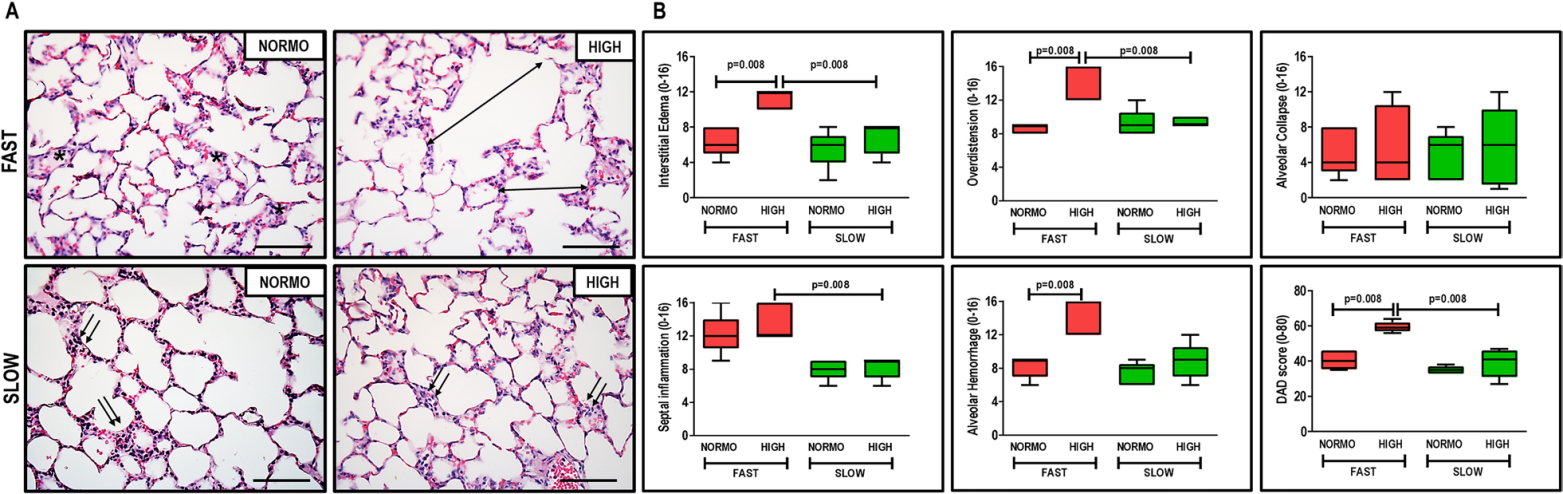
**A** representative photomicrographs (light microscopy) of lung parenchyma stained with hematoxylin–eosin. Photomicrographs are representative of data obtained from lung sections of 7 animals (original magnification, × 400). Bars = 100 μm. Note the presence of interstitial edema (asterisk), lung overdistension (double arrows), and alveolar-capillary damage (presence of neutrophils and erythrocytes in the alveolar septa) (two arrows). **B** diffuse alveolar damage (DAD) score; scores arithmetically averaged from two independent investigators representing injury from interstitial edema, overdistension, alveolar collapse, septal inflammation, and alveolar hemorrhage. Boxes show the interquartile (25–75%) range, whiskers encompass the range (minimum to maximum), and horizontal lines represent median values of seven animals/group. Rats received a standard (10 mL/kg/h, NORMO) or high (30 mL/kg/h, HIGH) volume of Ringer’s lactate. FAST: abrupt PEEP decrease from 9 to 3 cmH_2_O; SLOW: gradual PEEP decrease (0.2 cmH_2_O/min) from 9 to 3 cmH_2_O

In animals treated with abrupt PEEP deflation, high fluid compared to standard fluid administration resulted in increased DAD score due to less interstitial edema, overdistension, and alveolar hemorrhage (Fig. 2). Abrupt compared with gradual PEEP deflation, when combined with high fluid administration, was associated with high DAD score due to reduced interstitial edema, overdistension, septal inflammation. No significant differences were observed in the degree of alveolar collapse among groups. Ultrastructural analysis of lung parenchyma showed greater interstitial edema in HIGH compared to NORMO animals, independent of PEEP decrease rate. Basement membrane injury, extracellular matrix damage, type II epithelial dell damage, and endothelial cell damage scores were greater in the FAST groups than in the SLOW groups, regardless of fluid status (Additional file 3: Table S2 and Fig. S2).

**Fig. 3:**
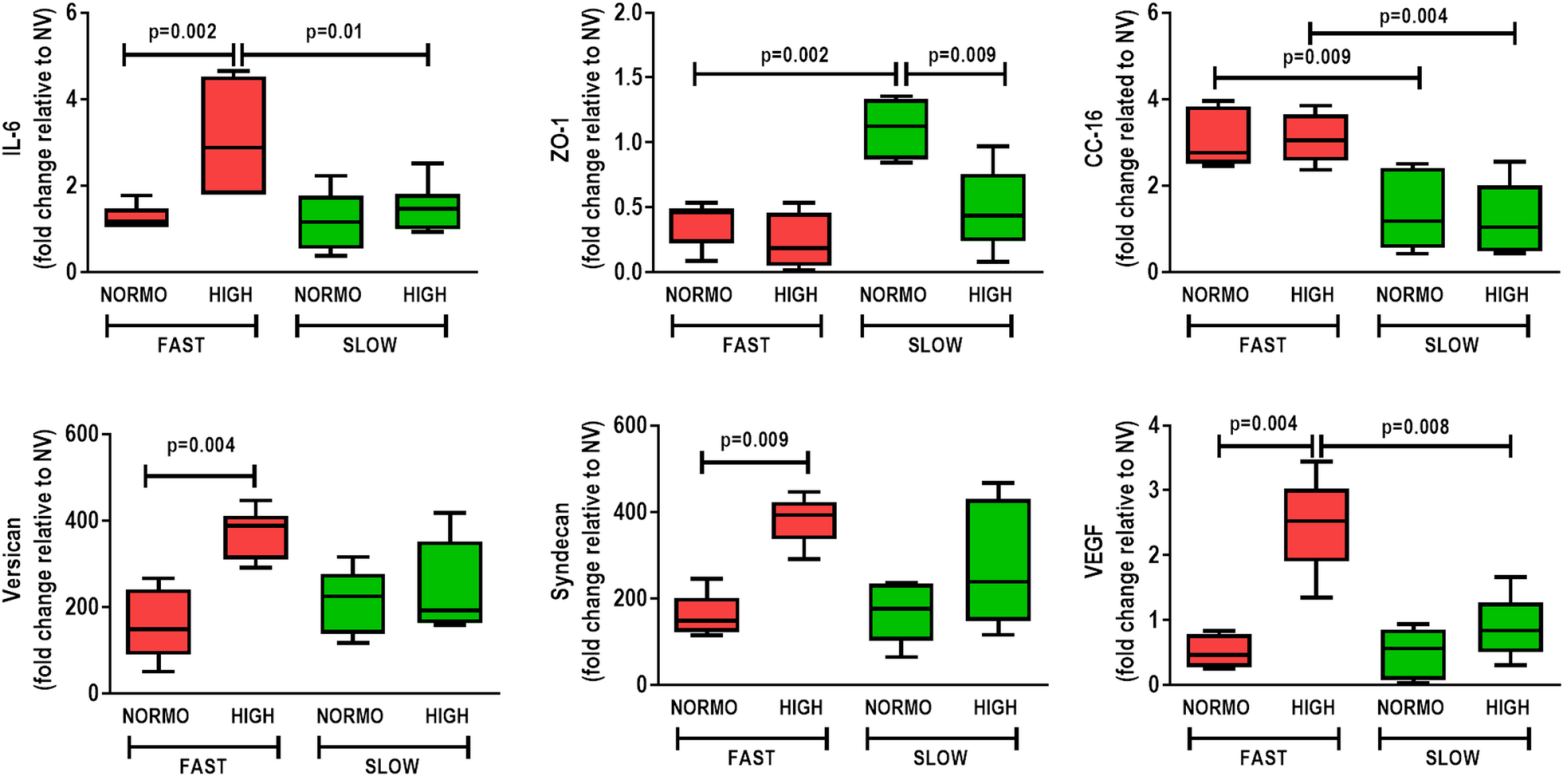
Expression of biomarkers associated with inflammation [interleukin (IL)-6], tight junctions [zonula occludens (ZO)-1], epithelial cell damage [club cell protein (CC)-16], extracellular matrix remodeling (versican, syndecan-1), and endothelial cell damage [(vascular endothelial growth factor (VEGF)]. Relative gene expression was calculated as a ratio of the average gene expression levels compared with the reference gene (acidic ribosomal phosphoprotein P0, *36B4*) and expressed as fold change relative to non-ventilated animals (NV). Boxes show the interquartile (25–75%) range, whiskers encompass the range (minimum to maximum), and horizontal lines represent median values of 7 animals/group. Rats received a standard (10 mL/kg/h, NORMO) or high (30 mL/kg/h, HIGH) volume of Ringer’s lactate. FAST: abrupt PEEP decrease from 9 to 3 cmH_2_O; SLOW: gradual PEEP decrease (0.2 cmH_2_O/min) from 9 to 3 cmH_2_O

The combination of abrupt PEEP decrease and high fluid administration led to increased gene expressions of IL-6, versican, syndecan-1, and VEGF. Under high fluid conditions, gradual PEEP decrease resulted in reduced IL-6 and VEGF. Gene expression of ZO-1 was greater in the group treated with standard fluid administration plus gradual PEEP decrease compared to other groups, suggesting epithelial cell preservation. CC-16 was overexpressed in the abrupt PEEP decrease groups, regardless of fluid status (Fig. 3).

**Fig. 4:**
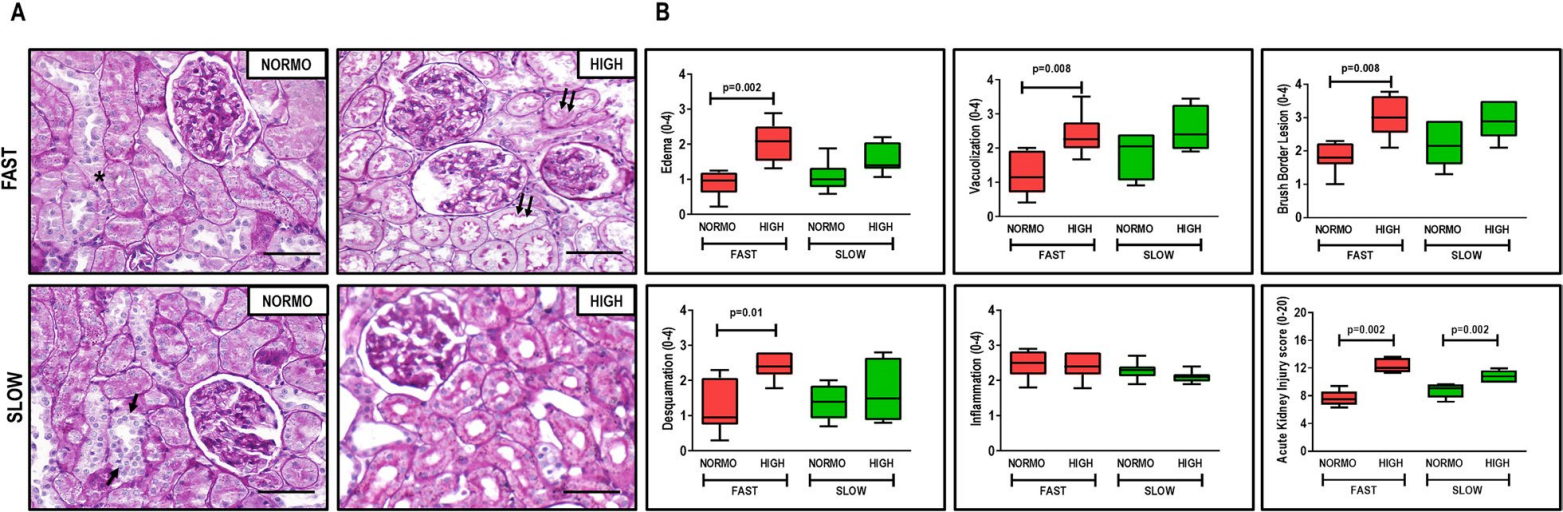
**A** representative photomicrographs (light microscopy) of kidney parenchyma stained with hematoxylin–eosin. Photomicrographs are representative of data obtained from kidney sections of 7 animals (original magnification, × 400). Bars = 100 μm. Note the presence of interstitial edema (asterisk), brush border lesion (two arrows), and cellular desquamation (one arrow). **B** kidney injury score; scores arithmetically averaged from two independent investigators representing injury from interstitial edema, vacuolization, brush border lesion, desquamation, and inflammation are shown. Boxes show the interquartile (25–75%) range, whiskers encompass the range (minimum to maximum), and horizontal lines represent median values of 7 animals/group

**Fig. 5:**
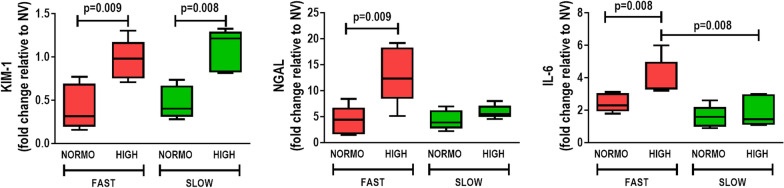
Expression of biomarkers associated with renal tubular epithelial cell damage [kidney injury molecule (KIM)-1 and neutrophil gelatinase-associated lipocalin (NGAL)] and inflammation [interleukin (IL)-6]. Relative gene expression was calculated as a ratio of the average gene expression levels compared with the reference gene (acidic ribosomal phosphoprotein P0, *36B4*) and expressed as fold change relative to non-ventilated animals (NV). Boxes show the interquartile (25–75%) range, whiskers encompass the range (minimum to maximum), and horizontal lines represent median values of 7 animals/group. Rats received a standard (10 mL/kg/h, NORMO) or high (30 mL/kg/h, HIGH) volume of Ringer’s lactate. FAST: abrupt PEEP decrease from 9 to 3 cmH_2_O; SLOW: gradual PEEP decrease (0.2 cmH_2_O/min) from 9 to 3 cmH_2_O

High fluid administration was associated with higher kidney damage score (Fig. 4) and IL-6 expression (Fig. 5), independent of the rate of PEEP decrease. After abrupt PEEP decrease, interstitial edema, tubular cell vacuolization, brush border derangement in proximal tubular epithelia, and tubular cell desquamation (Fig. 4), as well as KIM-1, NGAL, and IL-6 gene expressions, were higher in the HIGH than in the NORMO animals (Fig. 5). In the gradual PEEP decrease groups, KIM-1 expression was greater in HIGH than in NORMO.

## Discussion

In the model of mild-moderate ARDS used herein, we found that animals treated with high fluids and abrupt PEEP decrease exhibited greater diffuse alveolar damage and higher expression of genes associated with lung inflammation and endothelial cell damage compared to the other groups. Abrupt PEEP reduction, regardless of fluid status, led to greater epithelial cell damage and pulmonary arterial pressure (as indicated by the PAT/PET ratio). Standard fluid administration combined with gradual PEEP reduction better preserved alveolar epithelial cells. Acute kidney injury score and gene expression of kidney injury molecule-1 was higher in high compared to standard fluid administration, during both abrupt and gradual PEEP decrease.

Experimental ARDS induced by intratracheal instillation of endotoxin (first hit) [24] resulted in changes in lung mechanics and histology that resembled human ARDS and is in agreement with the American Thoracic Society committee recommendations [24]. Therefore, the definition of mild, moderate and severe ARDS was assigned based on morphological and functional changes and not designated according to PaO_2_/FiO_2_ at PEEP level ≥ 5 cmH_2_O.

Animals were then randomized to receive different mechanical ventilation strategies and fluids as a second hit. Among the crystalloids, Ringer’s lactate was chosen since it avoids the hyperchloremic acidosis that predictably accompanies the use of saline [25]. The high rate of fluid infusion was based on previous pilot studies in animals with endotoxin-induced lung injury treated with different mechanical ventilation strategies, as well as standard and high-volume fluid administration. In this line, the fluid volumes of 7 mL versus 16 mL at FINAL kept MAP above 70 mmHg in our rats. The relative volume of fluids administered in rats differs from humans due to differences in lung pulmonary vessels (anatomy, size, and number). The initial PEEP level (9 cmH_2_O) was sufficient to keep the lungs fully open [15] and the animals alive for 1 h. Moreover, this PEEP level resembles that used in mechanically ventilated critical care patients (18 cmH_2_O), taking into account differences in transpulmonary pressures between humans and rats [26].

We abruptly decreased PEEP level across a pressure range that paralleled, but did not match, that of a previous study in rabbits [11]. Such decompressions may happen in practice during aggressive weaning or during accidental or intentional mechanical ventilator disconnection. PEEP = 9 cmH_2_O was applied for 30 min since, as based on previous pilot studies, it opens alveoli and keeps them homogeneously open. Moreover, 30 min is the minimum time needed to modify gene expression of the biomarkers of interest [26]. The lung tissue biomarkers measured in this study are indicative of increased inflammation (IL-6) [27], extracellular matrix damage (versican, syndecan) [28], endothelial cell injury (VEGF) [29], and tight junction integrity (ZO-1) [30]. The selected kidney biomarkers (KIM-1 and NGAL) are associated with renal proximal tubular injury [31] and severity of kidney disease [32].

The linkage of excessive strain, inflation energy, and cycling frequency with VILI is rather well established [6]. However, the parenchyma also absorbs some of the potential energy stored at end-inspiration, experiencing additional strains during the early part of deflation as the lung refolds and rearranges into its (proto-inspiratory) position. Experimental and clinical evidence provided by several groups suggests that slowing early expiration and regulating expiratory flow may reduce lung injury [33, 34]. While the exact mechanism underlying these intriguing and consistent observations remains obscure, the tacit inference has been that the explanation would center on the magnitude and distribution of the released parenchymal energy. That tissue energy load may influence both the airspace and vascular compartments.

Like the expiratory portion of the tidal cycle, the influence of vascular events has received relatively little consideration. The potential for very high capillary pressures and flows to cause stress fractures of the alveolar-capillary barrier provides a compelling explanation for the parenchymal hemorrhage that accompanies certain forms of cardiogenic pulmonary edema and the high cardiac output conditions of extreme athletic stress. A series of prior studies from one of our own groups demonstrated that the presence of high microvascular pressure gradients and flows may be an essential determinant of overt VILI expression for lungs ventilated with identical airspace and left atrial (pulmonary venous) pressures [2, 3]. The clear structural damage and endothelial damage that occurs in that setting may theoretically result (at least in part) from unmeasured shearing forces and energy released by blood flowing through a compressed and restricted vascular bed. Here, we showed that gradually decreasing the PEEP level promotes lung and kidney protection. We may infer that abruptly decreasing PEEP through a wide range may lead to profound hemodynamic changes as blood shifts from peripheral toward central vessels. If so, pulmonary endothelial cells and extracellular matrix are likely subjected to major biophysical challenges, such as supraphysiological levels of shear stress [35]. Endothelial cells have a variety of receptors, which sense flow and transmit mechanical signals through mechanosensitive signaling pathways to recipient molecules that lead to phenotypic and functional changes [36]. IL-6, versican, syndecan, and VEGF gene expressions in these experiments corroborate this hypothesis. VEGF, in conjunction with its receptor VEGFR2, has been shown to participate in the transmission of forces to intercellular junction protein complexes (VE-cadherin, β-catenin, and phosphatidylinositol 3-kinase to phosphorylate Akt and PECAM-1) [29]. On the other hand, the most protective strategy, namely NORMO-SLOW, led to high expression of ZO-1, which enhances endothelial bonding, preventing plasma leakage from vascular to alveolar spaces. Alternatively, altered perfusion might, in concept, influence the mechanical properties of the parenchyma sufficiently to accentuate micromechanical stresses during either half of the tidal cycle.

Data from the present study indicate that increased lung tissue damage results from an abrupt rather than a gradual PEEP release in the presence of an increased rate of fluid infusion. Abrupt transitioning caused the lung to exhibit histologic and molecular evidence of inflammation and endothelial trauma, which was not explained by the left ventricular loading mismatch reported by Katira et al. [11]. The differences between our findings may be related to the duration of high PEEP application, the nature of the model (healthy rabbits vs. pre-injured rats), or the magnitude of the sudden release to ZEEP (vs. 3 cmH_2_O). Rather, in our study the lung and right ventricle were adversely affected, whereas the echocardiographic ejection pattern of the left ventricle was not. Fluid loading produced the expected changes of tissue edema but affected the nature or severity of lung injury only inconsistently.

It should be noted that the mean airway pressures in the animals treated with a gradual PEEP decrease were somewhat higher than those in the abrupt PEEP decrease groups because of the intentional lingering of PEEP above the baseline value. One possibility to consider is that gradual decline may have promoted stress adaptation and a more even distribution of parenchymal strain. Simultaneously, however, a higher mean airway pressure resulted in lung overdistension which may increase pulmonary arterial pressure, with the potential to adversely affect the right ventricle—the opposite of our actual findings. The validity of such speculations regarding benefit of slower PEEP release, however, remains untested.

Kidney damage caused by endotoxin [20] is particularly sensitive to venous congestion. In this context, fluid overload can result in severe and sustained kidney injury [37]. In the model used for the present study, acute kidney injury score and gene expression of kidney injury molecule-1 were greater in HIGH than in NORMO fluid groups regardless of whether PEEP decrease was abrupt or gradual. One possibility is that differences in mechanotransduction induced by abrupt or gradual PEEP removal may yield less kidney damage than fluid increase itself. In fact, high rates of Ringer’s lactate administration appear to potentiate kidney damage by an as yet unconfirmed mechanism [38].

## Possible clinical implications

In ARDS, the combination of high PEEP and low tidal volume has been associated with reduced venous return, cardiac output, and pulmonary capillary inflow, while simultaneously increasing extravascular pressure. Consequently, lung edema may decline even in the presence of alveolar capillary damage [39]. During PEEP removal, hemodynamic instability and increased edema may occur, often presenting a challenge to correct management of the amount of fluids to be administered. According to the results of the present study: (1) PEEP should be reduced gradually, regardless of the amount of fluid administered, to avoid increasing pulmonary arterial hypertension; (2) Abrupt PEEP decreases in conjunction with high fluid therapy may promote lung inflammation and vascular damage; (3) Gradually reducing PEEP and administering conservative fluid volumes helps preserve the epithelium from further damage; and (4) To prevent kidney damage, liberal fluid administration should be avoided, regardless of the velocity of PEEP deflation. Data from rodent experiments afford only mechanistic insights into VILI and cannot be directly applied to clinical settings without reservation. Even though abrupt deflation (from 9 to 3 cmH_2_O in rats, approximating 18–6 cmH_2_O in humans) does not occur commonly in clinical practice, these data provide a proof of concept that VILI is influenced by interaction between the pace of PEEP decrease and the amount of fluids administered.

## Limitations

Some limitations of the present study must be acknowledged. Experiments were conducted in a small-animal model of mild-moderate ARDS and tested only one V_T_ and minute ventilation. These findings apply to these specific experimental circumstances and time intervals. The selected PEEP levels chosen for the PEEP transition were selected arbitrarily; 9 cmH_2_O approaches the upper limit of what rats can sustain for the intended duration of these experiments without life-threatening hemodynamic compromise, whereas 3 cmH_2_O is a well-tolerated level that may afford some level of lung protection against ventilation stress. The observation time was 1 h in order to avoid hemodynamic impairment, which might have introduced bias into our study. Accordingly, we did not assess protein levels of biological markers. However, this duration of mechanical ventilation was sufficient for gene expression of key markers of interest and corresponds to that used in a previous study [15]. Moreover, 1 rat hour approximates 27 human hours [41]. Echocardiography was performed 10 min after deflation, and thus we cannot rule out that a right-to-left ventricular mismatch may have occurred during or immediately after PEEP reduction. At FINAL, respiratory acidosis was observed in all groups and may mitigate lung damage [5, 40]. However, all groups presented the same level of pH and PaCO_2_, which may have thus affected mediators similarly.

## Conclusions

In the model of mild-moderate ARDS used herein, decreasing PEEP abruptly increased pulmonary arterial hypertension under background conditions of standard and high fluid administration. The combination of abrupt PEEP decrease and high fluid administration led to greater lung and kidney injury, inflammation, and endothelial cell damage. This information adds to the growing body of evidence that supports gradual transitioning of ventilatory patterns and warrants directing additional investigative effort into vascular and deflation issues that impact lung protection.

## Supplementary Information

**Additional file 1: Table S1.** Forward and reverse oligonucleotide sequences of target gene primers.

**Additional file 2: Figure S1.** Short-axis view of the left and right ventricles (upper panels) and pulmonary Doppler (lower panels).

**Additional file 3: Table S2.** Semiquantitative analysis of lung electron microscopy. **Figure S2.** Transmission electron photomicrographs of lung parenchyma.

## Data Availability

The datasets used and/or analyzed during the present study are available from the corresponding author on reasonable request.

## Abbreviations

ARDS: Acute respiratory distress syndrome
CC-16: Club cell protein 16
DAD: Diffuse alveolar damage
FiO_2_: Fraction of inspired oxygen
HR: Heart rate
IL: Interleukin
KIM: Kidney injury molecule
LPS: Lipopolysaccharide
LV: Left ventricle
MAP: Mean arterial pressure
NGAL: Neutrophil gelatinase-associated lipocalin
NV: Non-ventilated
PAT: Pulmonary acceleration time
PEEP: Positive end expiratory pressure
PET: Pulmonary ejection time
Pplat,_RS_: Respiratory system plateau pressure
RR: Respiratory rate
RT-PCR: Quantitative real-time reverse transcription polymerase chain reaction
RV: Right ventricle
RVCO: Right ventricular cardiac output
Ti/Ttot: Inspiratory time divided by duty cycle
V′_E_: Minute ventilation
VCV: Volume-controlled mode
VEGF: Vascular endothelial growth factor
VILI: Ventilator-induced lung injury
V_T_: Tidal volume
ZEEP: Zero end-expiratory pressure
ZO-1: Zona occludens-1
ΔP,_RS_: Respiratory system driving pressure
