# Supplementary material for: The impact of fluid status and decremental PEEP strategy on cardiac function and lung and kidney damage in mild-moderate experimental acute respiratory distress syndrome
Source: Respir Res. 2021 Jul 30;22:214. doi: 10.1186/s12931-021-01811-y (PMC8323327; doi:10.1186/s12931-021-01811-y)
Supplement: Supplementary file 1 — Additional file 1: Table S1. Forward and reverse oligonucleotide sequences of target gene primers. [file 12931_2021_1811_MOESM1_ESM.docx]

**Additional File 1**

**Table S1**: Forward and reverse oligonucleotide sequences of target gene primers

| **Gene** | **Primer** | **Primer sequences (5′-3′)** |
| --- | --- | --- |
| ***Lung*** | | |
| IL-6 | Forward | CTC CGC AAG AGA CTT CCA G |
|  | Reverse | CTC CTC TCC GGA CTT GTG A |
| ZO-1 | Forward | CAC CAC AGA CAT CCA ACC AG |
|  | Reverse | CAC CAA CCA CTC TCC CTT GT |
| CC-16 | Forward | GAT CGC CAT CAC AAT CAG TG |
|  | Reverse | GGT ATC CAC CAG CCT CTT CA |
| Versican | Forward | CCA CTT GGA AAG CCA GAC AT |
|  | Reverse | CCA GGG TCA CTT CTG TGG AT |
| Syndecan | Forward | GTT CCG CTG GTT TTG TTG TTT |
|  | Reverse | GAT GAA GGC TGT TCC CAG GTA |
| VEGF | Forward | CAG AAA GCC CAT GAA GTG GT |
|  | Reverse | ACA CAG GAC GGC TTG AAG AT |
| ***Kidney*** | | |
| KIM-1 | Forward | GAA GAA AAC AAT GGA TCA AGG GAT |
|  | Reverse | GGA GTG GAA ATG GCT CTA ATG AAC |
| NGAL | Forward | TCA AAG TCA CCC TGT ACG |
|  | Reverse | CCG TCT GTT CAG TTG TCA |
| IL-6 | Forward | CTC CGC AAG AGA CTT CCA G |
|  | Reverse | CTC CTC TCC GGA CTT GTG A |
| *36B4* | Forward | GGA TCA CTC AGG AGC AGG AG |
|  | Reverse | CTT GGC ACT CAA GAG GAA GG |

ZO-1, zonula occludens; KIM, kidney injury molecule-1; IL, interleukin-6; *36B4*, acidic ribosomal phosphoprotein P0.
