# Supplementary material for: The impact of fluid status and decremental PEEP strategy on cardiac function and lung and kidney damage in mild-moderate experimental acute respiratory distress syndrome
Source: Respir Res. 2021 Jul 30;22:214. doi: 10.1186/s12931-021-01811-y (PMC8323327; doi:10.1186/s12931-021-01811-y)
Supplement: Supplementary file 2 — Additional file 2: Figure S1. Short-axis view of the left and right ventricles (upper panels) and pulmonary Doppler (lower panels). [file 12931_2021_1811_MOESM2_ESM.docx]

**Additional File 2**


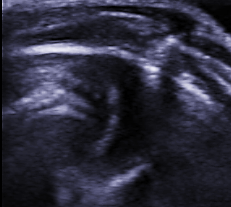


LV

RV


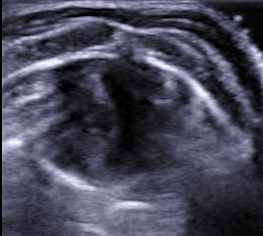


LV

RV


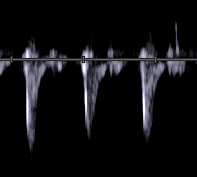

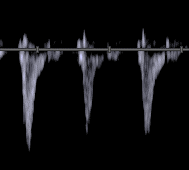


INITIAL FINAL

NORMO FAST

A

B

C

D


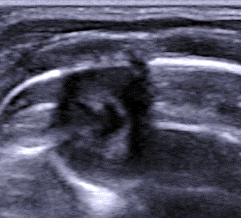


LV

RV


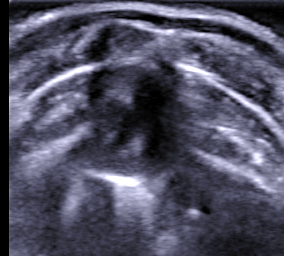


LV

RV


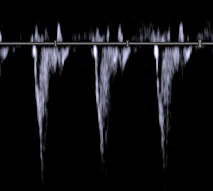

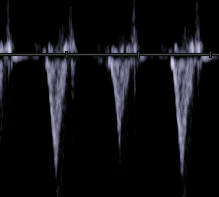


I

J

K

L

INITIAL FINAL

NORMO SLOW


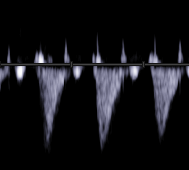

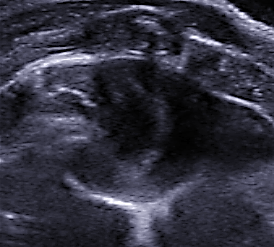


LV

RV


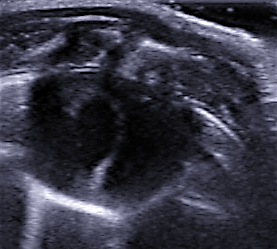


LV

RV


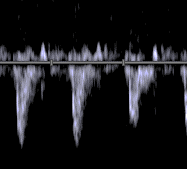


E

F

HIGH FAST

G

H


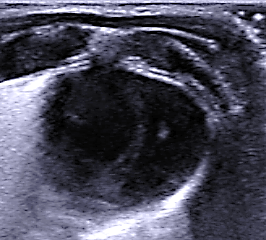

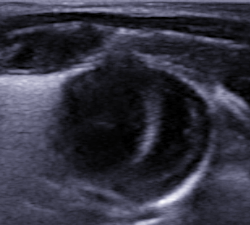

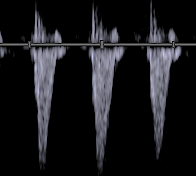

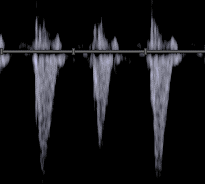


LV

RV

LV

RV

HIGH SLOW

M

N

O

P

**Fig. S1.** A, B, E, F, I, J, M and N. Short-axis view of the left and right ventricles (LV, RV). C, D, G, H, K, L, O and P. Pulmonary Doppler. The NORMO-FAST and HIGH-FAST groups exhibit significant enlargement of the RV at FINAL. Pulmonary Doppler shows a short acceleration time that remains at FINAL. The NORMO-SLOW group shows slight enlargement of the LV and RV, as well as in acceleration time at FINAL. Unlike the NORMO-SLOW group, the HIGH-SLOW group exhibited enlargement of LV at FINAL, which was accompanied by an increase in pulmonary flow.
