# Supplementary material for: The impact of fluid status and decremental PEEP strategy on cardiac function and lung and kidney damage in mild-moderate experimental acute respiratory distress syndrome
Source: Respir Res. 2021 Jul 30;22:214. doi: 10.1186/s12931-021-01811-y (PMC8323327; doi:10.1186/s12931-021-01811-y)
Supplement: Supplementary file 3 — Additional file 3: Table S2. Semiquantitative analysis of lung electron microscopy. Figure S2. Transmission electron photomicrographs of lung parenchyma. [file 12931_2021_1811_MOESM3_ESM.docx]

**Additional File 3**

**Table S2–** Semiquantitative analysis of lung electron microscopy

|  | **FLUIDS** | **PEEP** |  |
| --- | --- | --- | --- |
| **Interstitial edema** | **NORMO** | **FAST** | **2 (2 – 2)*** |
|  | **HIGH** |  | **4 (3 – 4)** |
|  | **NORMO** | **SLOW** | **1 (1 – 1)#** |
|  | **HIGH** |  | **3 (3 – 4)** |
| **Basement membrane injury** | **NORMO** | **FAST** | **3 (3 – 4)** |
|  | **HIGH** |  | **3 (3 – 4)** |
|  | **NORMO** | **SLOW** | **1 (1 – 2)#** |
|  | **HIGH** |  | **2 (1 – 2)*** |
| **Extracellular matrix**  **damage** | **NORMO** | **FAST** | **2 (2 – 3)** |
|  | **HIGH** |  | **3 (3 – 4)** |
|  | **NORMO** | **SLOW** | **1 (1 – 2)#** |
|  | **HIGH** |  | **1 (1 – 2)*** |
| **Type II epithelial cell damage** | **NORMO** | **FAST** | **3 (3 – 4)** |
|  | **HIGH** |  | **3 (2 – 3)** |
|  | **NORMO** | **SLOW** | **1 (1 – 2)#** |
|  | **HIGH** |  | **1 (1 – 2)*** |
| **Endothelial cell damage** | **NORMO** | **FAST** | **3 (3 – 4)** |
|  | **HIGH** |  | **3 (3 – 3)** |
|  | **NORMO** | **SLOW** | **1 (1 – 2)#** |
|  | **HIGH** |  | **2 (2 – 2)*** |

Pathologic findings were graded on a five-point, semiquantitative, severity-based scoring system: 0=normal lung parenchyma, 1=changes in 1 to 25% of examined tissue, 2=26 to 50% of examined tissue, 3=51 to 75% of examined tissue, and 4=76 to 100% of examined tissue. Values are median (interquartile range) of 7 animals per group. NORMO and HIGH: administration of Ringer lactate at 10 mL/kg/h and 30 mL/kg/h, respectively. FAST: abrupt PEEP decrease from 9 to 3 cmH_2_O. SLOW: gradual PEEP decrease (0.2 cmH_2_O/min) from 9 to 3 cmH_2_O. *Significantly different from HIGH-FAST (p<0.0125). #Significantly different from NORMO-FAST (p<0.0125).


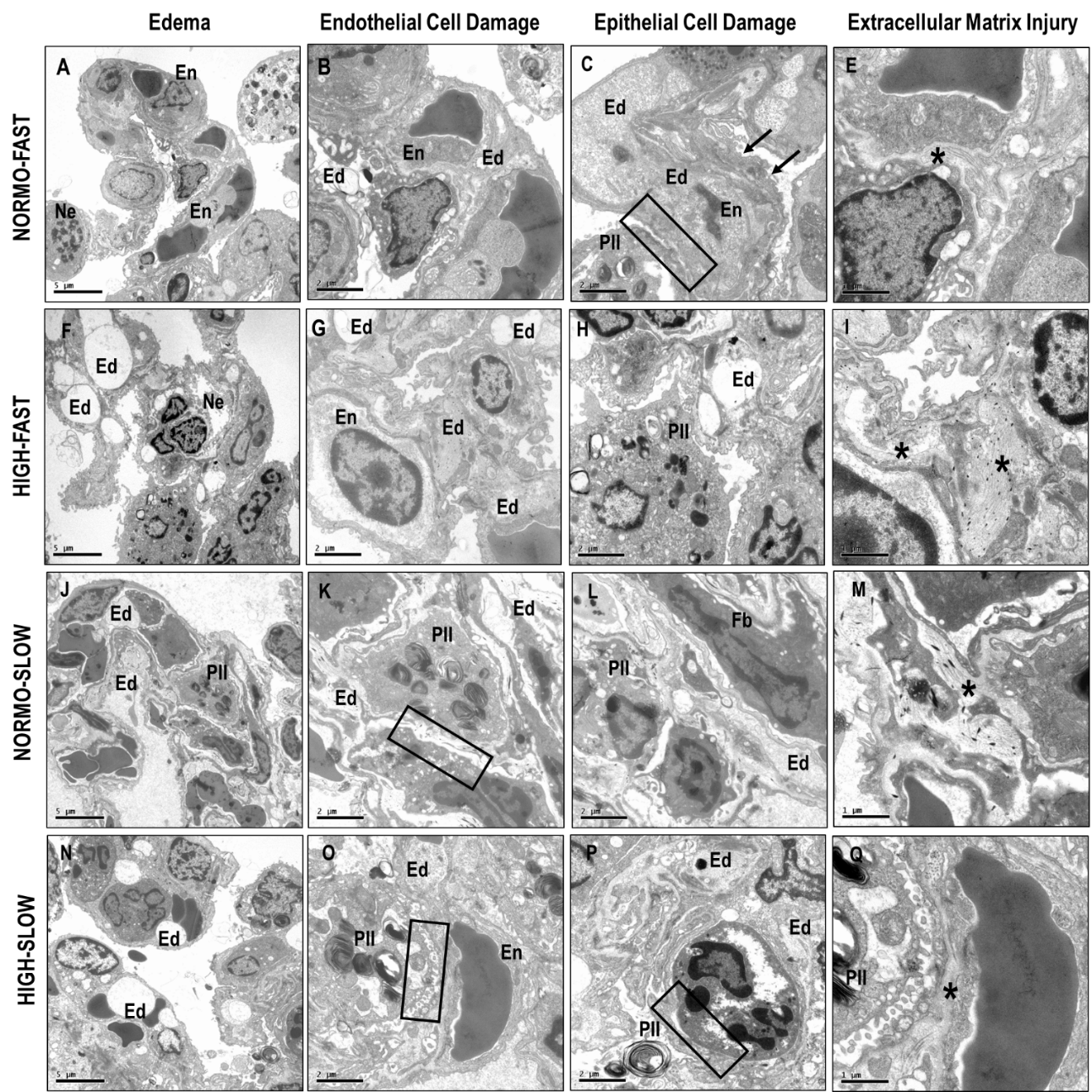


**Fig. S2. Transmission electron photomicrographs of lung parenchyma.** Ultrastructural features of the alveolar–capillary barrier at different combinations of fluid volume and PEEP decrement. In the NORMO-FAST group, edema along the alveolar septa is less prominent (A, B) but there is more endothelial cell apoptosis (B), type II epithelial cell detachment, and irregularity of the basement membrane (C), as well as less collagen fiber disarrangement (D). The HIGH-FAST group shows increased neutrophil counts (E), prominent edema in the form of bubbles along the alveolar septa (E, F), endothelial cell apoptosis (F), fragmentation of the lamellar bodies in type II epithelial cells (PII), and disarrangement of collagen fibers (H). In the NORMO-SLOW group, there is substantial attenuation of edema compared with other groups (I), relatively preserved integrity of endothelial cells (K), basement membrane (K), and lamellar bodies of type II epithelial cells (K), and less disarrangement of collagen fibers, which were also better distributed along the alveolar septa (L). The HIGH-SLOW group also presented both interstitial and alveolar edema. Note the presence of bubbles along the alveolar septa (M, N). However, the degree of damage to endothelial cells (N), basement membrane (N) and type II epithelial cells (O), as well as disarrangement of collagen fibers (P), was substantially reduced compared to FAST groups. Ne, neutrophils; Ed, edema; En, endothelium; Square, basement membrane; PII, type II epithelial cells. *collagen fibers.
